# IPUMS Multigenerational Longitudinal Panel

**DOI:** 10.23889/ijpds.v9i5.2870

**Published:** 2024-09-18

**Authors:** Steven Ruggles, Jonas Helgertz, Catherine Fitch

**Affiliations:** 1 University of Minnesota, Twin Cities; 2 Lund University

Over the past five years, the IPUMS Multigenerational Longitudinal Panel (MLP) has constructed a massive longitudinal population panel by linking together American censuses, surveys, administrative sources, and vital records spanning the period from 1850 to the present. MLP is designed as a general-purpose resource for studying longitudinal processes and long-run social and economic change. By linking individuals across generations from birth through death using data from multiple sources, MLP enables reproducible research on shifting patterns of life course patterns and intergenerational processes. The large scale of the MLP database allows researchers both to study particular communities and small dispersed populations and to conduct big studies spanning many places and periods. Data that allow investigators to examine simultaneously the broad sweep of time and fine spatial detail will yield new insights into ongoing transformations of demographic behavior.

The presentation will describe the creation of the IPUMS MLP database and the current MLP linking strategy. We then detail our plans for expansion and improvement of MLP over the next five years, including the incorporation of additional data sources, the development of a “linkage hub” to connect MLP with other major record linkage efforts, and the refinement of our technology and dissemination efforts. We will conclude by describing a few early examples of MLP-based research.

